# Draft genome sequences of 180 bacterial strains recovered from ground beef in retail markets in Fort Collins, Colorado

**DOI:** 10.1128/mra.00226-26

**Published:** 2026-04-20

**Authors:** Chloe Kane, Ifigenia Geornaras, Peipei Zhang

**Affiliations:** 1 Department of Animal Sciences, Colorado State University 728642, Fort Collins, Colorado, USA; University of Maryland Baltimore, Baltimore, Maryland, USA

**Keywords:** ground beef, spoilage-associated bacteria, air packaging, *Pseudomonas*, lactic acid bacteria

## Abstract

Limited studies have characterized bacteria contributing to raw meat spoilage at the species/strain level. We sequenced 180 bacterial strains recovered from aerobically packaged ground beef. The information from this study will help us better understand the composition of bacterial communities associated with ground beef spoilage and the related genetic factors.

## ANNOUNCEMENT

Bacteria are one of the primary drivers of raw meat spoilage and food waste. Biological activities of bacteria often vary among species or even strains (1). However, there is a limited understanding of the species/strain-level composition of bacterial communities contributing to meat spoilage. This study recovered and sequenced bacterial strains associated with ground beef spoilage.

Aerobically packaged ground beef was purchased from grocery stores in Fort Collins, Colorado, and stored at 3°C until 4 days past the date on the package label (i.e., use-by, freeze-by, sell-by, or sell-through dates, depending on the store). Beef (100 g) from each package was mixed with 300 mL of peptone water (0.1%, w/v, Bacto, Life Technologies), mechanically pummeled, and serially diluted. Aliquots (100 µL) of the 10^−3^, 10^−4^, and 10^−5^ dilutions were spread onto tryptic soy agar (TSA; Difco, BD), and the plates were incubated at 25°C for 3 days. For each sample, five colonies were randomly selected from TSA plates with >5 colonies and purified by streaking. Purified isolates were separately inoculated into tryptic soy broth (Difco, BD) and incubated at 25°C until stationary growth phase. DNA of each culture was extracted using the DNeasy Blood and Tissue Kit (Qiagen) by following the manufacturer’s instructions for gram-positive bacteria. Random amplified polymorphic DNA (RAPD) polymerase chain reaction (PCR) (2) was performed to differentiate clonal isolates from each beef sample. DNA of unique strains was sent to Sailgene Technology (USA) Inc. for whole genome sequencing. The Hieff NGS OnePot Pro DNA Library Prep Kit V4 was used for sequencing library construction, with an enzymatic reaction at 37°C for 10 min during the DNA fragmentation step, followed by selection of DNA fragments of 450–500 bp. Paired-end sequencing (150 bp) was performed using the DNBSEQ-T7 platform.

For bioinformatics analysis, default parameters were used for all software unless otherwise specified. Specifically, Fastp (v1.0.1) (3) was used to remove adapters and sequencing reads with low quality and short length (≤50 bases). FastQC (v0.12.1) (4) and MultiQC (v1.33) (5) were used to examine the quality of sequencing reads before and after trimming. Genomes were assembled using SPAdes (v4.2.0) (6) with kmers set at 21, 33, 55, 77, and 99 bp. A Python script was used to remove contigs with <500 bp and low coverage (<10x) (7). GTDB-Tk (v2.6.1) (8) with reference database R226 was used to determine the species identity of each genome, and an average nucleotide identity of 95% was used as a threshold for species delineation. The genomes were annotated by the NCBI Prokaryotic Genome Annotation Pipeline (v6.10) (9–11).

A total of 180 unique bacterial strains were sequenced. Sequencing data and genome characteristics are summarized in Table 1. Most of the strains were identified as *Pseudomonas* (*n* = 110) or lactic acid bacteria (*n* = 49), including *Carnobacterium*, *Pseudolactococcus*, *Leuconostoc*, *Latilactobacillus*, and *Enterococcus*. It should be recognized that the bacteria were recovered from aerobic conditions and did not include anaerobes that may also play a role in ground beef spoilage.

**TABLE 1:** The genome sequences of 180 bacterial strains recovered from ground beef

Strain ID	Species	GC content (%)	Assembly size (bp)	Total number of genes	Number of sequencing reads	Genome coverage	Number of contigs	N50 (Kbp)	Accession number	
									Sequence read archive	Genome
B124	*Acinetobacter harbinensis*	41.2	3,040,284	2,920	7,209,752	356	79	91.4	SRR37195489	JBUZUP000000000
B423	*Acinetobacter harbinensis*	40.9	3,229,245	3,129	9,414,286	437	61	163.9	SRR37195377	JBUZTM000000000
B235	*Brochothrix thermosphacta*	36.1	2,603,906	2,556	10,173,424	586	35	210.4	SRR37195476	JBUZUD000000000
B321	*Brochothrix thermosphacta*	36.2	2,604,455	2,555	11,714,982	675	34	210.4	SRR37195472	JBUZTZ000000000
B324	*Brochothrix thermosphacta*	36.2	2,605,207	2,568	9,079,428	523	36	210.4	SRR37195470	JBUZTX000000000
C132	*Brochothrix thermosphacta*	36.3	2,524,542	2,494	9,634,378	572	18	257.8	SRR37195525	JBUZRW000000000
C323	*Brochothrix thermosphacta*	36.3	2,503,408	2,462	10,296,618	617	31	368.7	SRR37195501	JBUZRA000000000
C325	*Brochothrix thermosphacta*	36.3	2,510,179	2,413	9,765,536	584	18	508.3	SRR37195499	JBUZQY000000000
D222	*Brochothrix thermosphacta*	36.2	2,594,003	2,567	13,046,738	754	17	516.8	SRR37195446	JBUZQF000000000
D234	*Brochothrix thermosphacta*	36.2	2,594,758	2,578	13,254,696	766	17	516.8	SRR37195443	JBUZQC000000000
E1211	*Brochothrix thermosphacta*	36.2	2,555,472	2,475	9,129,114	536	24	219.0	SRR37195442	JBUZQB000000000
E1213	*Brochothrix thermosphacta*	36.3	2,580,214	2,500	10,697,918	622	18	400.0	SRR37195440	JBUZQA000000000
A222	*Carnobacterium divergens*	35.0	2,671,496	2,593	8,764,036	492	16	390.4	SRR37195441	JBUZVF000000000
A331	*Carnobacterium divergens*	35.0	2,694,431	2,634	11,700,312	651	32	216.4	SRR37195385	JBUZUW000000000
B323	*Carnobacterium divergens*	35.1	2,587,654	2,507	9,412,160	546	29	240.1	SRR37195471	JBUZTY000000000
B523	*Carnobacterium divergens*	35.1	2,638,643	2,585	9,427,822	536	30	216.4	SRR37195364	JBUZTA000000000
C235	*Carnobacterium divergens*	35.1	2,588,145	2,507	10,659,052	618	30	240.1	SRR37195508	JBUZRG000000000
D111	*Carnobacterium divergens*	35.0	2,703,991	2,635	8,486,876	471	33	353.2	SRR37195461	JBUZQT000000000
D112	*Carnobacterium divergens*	35.0	2,704,799	2,637	12,145,348	674	36	353.2	SRR37195460	JBUZQS000000000
D211	*Carnobacterium divergens*	35.4	2,761,128	2,703	9,312,954	506	20	294.2	SRR37195450	JBUZQJ000000000
D212	*Carnobacterium divergens*	35.4	2,666,289	2,598	13,319,546	749	16	418.0	SRR37195449	JBUZQI000000000
F111	*Carnobacterium divergens*	35.0	2,670,540	2,612	8,766,410	492	43	284.3	SRR37195398	JBUZPC000000000
F115	*Carnobacterium divergens*	35.4	2,739,638	2,684	12,822,204	702	16	434.3	SRR37195397	JBUZPB000000000
A134	*Carnobacterium maltaromaticum*	34.4	3,553,807	3,357	8,869,438	374	41	233.2	SRR37195356	JBUZVM000000000
A135	*Carnobacterium maltaromaticum*	34.3	3,672,929	3,453	8,985,014	367	55	229.8	SRR37195425	JBUZVL000000000
A221	*Carnobacterium maltaromaticum*	34.3	3,478,237	3,260	10,937,422	472	21	553.6	SRR37195452	JBUZVG000000000
A332	*Carnobacterium maltaromaticum*	34.3	3,576,483	3,371	9,804,956	411	44	363.2	SRR37195384	JBUZUV000000000
B432	*Carnobacterium maltaromaticum*	34.3	3,541,085	3,310	8,563,448	363	20	563.8	SRR37195374	JBUZTJ000000000
C123	*Carnobacterium maltaromaticum*	34.4	3,392,866	3,154	11,803,382	522	17	499.1	SRR37195416	JBUZSA000000000
C231	*Carnobacterium maltaromaticum*	34.4	3,556,748	3,324	11,777,124	497	23	378.2	SRR37195511	JBUZRJ000000000
D114	*Carnobacterium maltaromaticum*	34.4	3,579,791	3,358	10,606,294	444	36	509.7	SRR37195459	JBUZQR000000000
D233	*Carnobacterium maltaromaticum*	34.5	3,535,445	3,343	12,687,604	538	30	303.8	SRR37195444	JBUZQD000000000
F124	*Carnobacterium maltaromaticum*	34.4	3,586,911	3,412	10,185,706	426	28	314.9	SRR37195395	JBUZOZ000000000
B133	*Enterococcus faecalis*	37.4	3,092,183	3,021	8,243,440	400	25	465.9	SRR37195486	JBUZUM000000000
B313	*Enterococcus faecalis*	37.5	2,960,517	2,900	13,028,560	660	32	604.5	SRR37195474	JBUZUB000000000
B314	*Enterococcus faecalis*	37.5	2,804,291	2,738	9,063,756	485	42	262.0	SRR37195473	JBUZUA000000000
E131	*Enterococcus faecalis*	37.6	2,862,058	2,758	12,023,538	630	12	606.3	SRR37195437	JBUZPX000000000
B311	*Escherichia coli*	50.7	4,873,972	4,727	11,921,500	367	69	248.0	SRR37195475	JBUZUC000000000
E132	*Escherichia coli*	50.4	4,990,683	4,842	8,479,018	255	57	271.4	SRR37195436	JBUZPW000000000
E235	*Escherichia coli*	50.4	4,990,627	4,842	7,739,092	233	56	271.4	SRR37195411	JBUZPO000000000
E331	*Escherichia coli*	50.4	4,991,328	4,842	9,876,620	297	56	271.4	SRR37195400	JBUZPE000000000
C233	*Ewingella* sp.	52.7	4,926,199	4,556	11,209,350	341	23	2693.5	SRR37195510	JBUZRI000000000
B513	*Hafnia paralvei*	48.3	4,859,042	4,494	9,321,864	288	26	505.2	SRR37195369	JBUZTE000000000
E133	*Hafnia paralvei*	48.3	4,858,434	4,492	13,038,328	403	23	505.2	SRR37195435	JBUZPV000000000
E211	*Latilactobacillus sakei*	40.9	2,044,115	2,120	12,304,204	903	58	128.4	SRR37195433	JBUZPT000000000
E213	*Latilactobacillus sakei*	40.9	2,044,293	2,114	10,602,570	778	58	128.4	SRR37195432	JBUZPS000000000
D132	*Leuconostoc gasicomitatum*	36.7	1,861,781	1,880	13,038,384	1050	31	147.7	SRR37195454	JBUZQM000000000
D133	*Leuconostoc gasicomitatum*	36.7	1,861,574	1,878	12,550,652	1011	31	147.7	SRR37195453	JBUZQL000000000
B511	*Leuconostoc mesenteroides*	37.6	1,873,331	1,871	10,019,064	802	22	319.9	SRR37195371	JBUZTG000000000
A112	*Pseudolactococcus carnosus*	38.7	2,193,554	2,144	7,697,628	526	34	118.0	SRR37195529	JBUZVT000000000
A122	*Pseudolactococcus carnosus*	38.5	2,297,503	2,273	10,145,024	662	31	165.4	SRR37195480	JBUZVQ000000000
A131	*Pseudolactococcus carnosus*	38.3	2,471,021	2,443	8,603,728	522	56	134.7	SRR37195378	JBUZVO000000000
A323	*Pseudolactococcus carnosus*	38.8	2,137,181	2,085	8,032,774	564	29	137.9	SRR37195387	JBUZUY000000000
A325	*Pseudolactococcus carnosus*	38.7	2,151,240	2,106	7,829,016	546	61	59.9	SRR37195386	JBUZUX000000000
B121	*Pseudolactococcus carnosus*	38.6	2,266,300	2,213	9,720,244	643	20	1187.3	SRR37195492	JBUZUR000000000
B434	*Pseudolactococcus carnosus*	38.6	2,232,578	2,185	11,212,696	753	37	169.8	SRR37195373	JBUZTI000000000
B514	*Pseudolactococcus carnosus*	38.8	2,231,425	2,158	9,448,656	635	29	187.5	SRR37195368	JBUZTD000000000
C121	*Pseudolactococcus carnosus*	38.8	2,102,687	2,050	8,494,232	606	62	60.5	SRR37195418	JBUZSC000000000
D134	*Pseudolactococcus carnosus*	38.5	2,338,678	2,348	13,131,870	842	43	105.1	SRR37195451	JBUZQK000000000
D213	*Pseudolactococcus carnosus*	38.7	2,177,400	2,135	10,127,236	698	55	119.9	SRR37195448	JBUZQH000000000
E231	*Pseudolactococcus carnosus*	38.4	2,420,306	2,413	12,486,964	774	32	179.9	SRR37195431	JBUZPR000000000
E232	*Pseudolactococcus carnosus*	38.6	2,232,511	2,207	11,446,088	769	30	151.6	SRR37195413	JBUZPQ000000000
F131	*Pseudolactococcus carnosus*	38.5	2,355,132	2,347	12,779,274	814	38	211.6	SRR37195394	JBUZOY000000000
F134	*Pseudolactococcus carnosus*	38.3	2,385,615	2,365	13,060,044	821	34	239.4	SRR37195393	JBUZOX000000000
A121	*Pseudolactococcus paracarnosus*	38.1	2,314,640	2,301	8,487,020	550	47	99.2	SRR37195491	JBUZVR000000000
A132	*Pseudolactococcus paracarnosus*	38.1	2,297,072	2,307	10,079,356	658	41	155.2	SRR37195367	JBUZVN000000000
A321	*Pseudolactococcus paracarnosus*	38.0	2,336,242	2,354	9,012,386	579	38	155.2	SRR37195388	JBUZUZ000000000
C122	*Pseudolactococcus paracarnosus*	38.0	2,336,346	2,352	10,562,154	678	38	155.2	SRR37195417	JBUZSB000000000
A223	*Pseudomonas bubulae*	57.8	5,304,918	4,931	8,794,478	249	26	444.9	SRR37195414	JBUZVE000000000
B221	*Pseudomonas bubulae*	58.1	5,282,080	4,923	10,270,344	292	23	574.9	SRR37195482	JBUZUI000000000
B234	*Pseudomonas bubulae*	58.1	5,429,196	5,082	10,255,510	283	36	600.1	SRR37195477	JBUZUE000000000
B335	*Pseudomonas bubulae*	58.3	5,115,133	4,753	9,660,100	283	54	347.5	SRR37195464	JBUZTS000000000
B411	*Pseudomonas bubulae*	57.9	5,571,540	5,189	8,075,708	217	36	470.3	SRR37195463	JBUZTR000000000
B415	*Pseudomonas bubulae*	57.8	5,738,739	5,408	10,402,620	272	24	515.4	SRR37195381	JBUZTP000000000
B512	*Pseudomonas bubulae*	58.3	5,210,055	4,909	8,881,602	256	63	447.9	SRR37195370	JBUZTF000000000
B521	*Pseudomonas bubulae*	58.1	5,281,924	4,922	12,752,524	362	24	574.9	SRR37195365	JBUZTB000000000
B535	*Pseudomonas bubulae*	58.2	5,210,269	4,841	9,708,148	279	38	454.7	SRR37195358	JBUZSU000000000
B614	*Pseudomonas bubulae*	58.1	5,280,933	4,925	9,024,864	256	24	488.8	SRR37195353	JBUZSQ000000000
B625	*Pseudomonas bubulae*	58.1	5,282,056	4,924	10,670,406	303	27	489.3	SRR37195429	JBUZSM000000000
C334	*Pseudomonas bubulae*	58.0	5,241,141	4,875	12,285,538	352	16	731.5	SRR37195462	JBUZQU000000000
E1215	*Pseudomonas deceptionensis*	58.2	5,386,892	5,041	13,208,734	368	25	841.8	SRR37195438	JBUZPY000000000
A235	*Pseudomonas fragi*	59.3	5,270,519	4,891	9,859,832	281	40	308.4	SRR37195389	JBUZVA000000000
B114	*Pseudomonas fragi*	59.5	5,188,605	4,798	10,199,780	295	43	263.9	SRR37195493	JBUZUS000000000
B122	*Pseudomonas fragi*	59.5	5,187,026	4,797	11,003,374	318	40	263.9	SRR37195490	JBUZUQ000000000
B612	*Pseudomonas fragi*	59.2	5,236,387	4,944	9,879,596	283	102	122.5	SRR37195355	JBUZSS000000000
C221	*Pseudomonas fragi*	58.7	5,714,373	5,401	12,976,690	341	103	145.4	SRR37195516	JBUZRO000000000
C223	*Pseudomonas fragi*	58.7	5,715,916	5,400	11,362,644	298	104	145.4	SRR37195514	JBUZRM000000000
C224	*Pseudomonas fragi*	58.7	5,716,726	5,403	10,342,082	271	103	143.0	SRR37195513	JBUZRL000000000
C234	*Pseudomonas fragi*	59.1	5,177,267	4,725	12,853,780	372	31	407.2	SRR37195509	JBUZRH000000000
C333	*Pseudomonas fragi*	59.1	5,294,591	4,888	12,358,908	350	29	496.8	SRR37195496	JBUZQV000000000
B632	*Pseudomonas haemolytica*	59.8	6,234,022	5,743	6,804,844	164	25	640.0	SRR37195427	JBUZSK000000000
B633	*Pseudomonas haemolytica*	59.8	6,328,659	5,857	11,834,638	281	31	622.5	SRR37195426	JBUZSJ000000000
B634	*Pseudomonas haemolytica*	59.8	6,233,204	5,744	11,253,762	271	27	622.4	SRR37195424	JBUZSI000000000
B524	*Pseudomonas helleri*	58.2	5,617,011	5,121	11,130,996	297	41	330.1	SRR37195363	JBUZSZ000000000
C133	*Pseudomonas hygromyciniae*	60.3	6,283,103	5,731	13,243,200	316	32	636.6	SRR37195524	JBUZRV000000000
B613	*Pseudomonas iridis*	58.7	6,312,022	5,843	9,265,134	220	34	329.6	SRR37195354	JBUZSR000000000
A111	*Pseudomonas kulmbachensis*	57.4	5,384,329	4,958	8,694,796	242	22	591.9	SRR37195530	JBUZVU000000000
A115	*Pseudomonas kulmbachensis*	57.2	5,478,159	5,055	10,005,756	274	23	612.0	SRR37195390	JBUZVS000000000
A211	*Pseudomonas kulmbachensis*	57.1	5,447,152	5,038	8,521,746	235	37	419.1	SRR37195528	JBUZVK000000000
C111	*Pseudomonas kulmbachensis*	57.3	5,348,144	4,951	9,784,730	274	36	602.6	SRR37195423	JBUZSH000000000
C113	*Pseudomonas kulmbachensis*	57.0	5,424,959	5,057	9,547,744	264	83	260.4	SRR37195421	JBUZSF000000000
C115	*Pseudomonas kulmbachensis*	57.3	5,360,093	4,971	9,302,712	260	21	767.9	SRR37195419	JBUZSD000000000
C212	*Pseudomonas kulmbachensis*	57.4	5,346,587	4,945	12,772,838	358	33	731.0	SRR37195520	JBUZRR000000000
C215	*Pseudomonas kulmbachensis*	57.4	5,347,416	4,946	13,237,842	371	33	731.0	SRR37195518	JBUZRP000000000
D225	*Pseudomonas lactis*	59.9	6,783,346	6,304	11,222,608	248	109	150.6	SRR37195445	JBUZQE000000000
A231	*Pseudomonas lundensis*	58.7	4,906,338	4,536	9,142,620	280	43	462.0	SRR37195392	JBUZVC000000000
A233	*Pseudomonas lundensis*	58.5	5,124,472	4,756	7,659,816	224	37	304.5	SRR37195391	JBUZVB000000000
B213	*Pseudomonas lundensis*	58.6	4,969,716	4,611	8,770,056	265	58	248.9	SRR37195484	JBUZUK000000000
B215	*Pseudomonas lundensis*	58.6	4,968,835	4,611	9,545,812	288	55	273.0	SRR37195483	JBUZUJ000000000
B231	*Pseudomonas lundensis*	58.4	4,989,943	4,659	8,870,834	267	54	215.1	SRR37195481	JBUZUH000000000
B232	*Pseudomonas lundensis*	58.3	5,450,251	5,129	9,007,570	248	64	284.5	SRR37195479	JBUZUG000000000
B331	*Pseudomonas lundensis*	58.6	4,969,561	4,613	9,101,628	275	57	248.9	SRR37195468	JBUZTW000000000
B515	*Pseudomonas lundensis*	58.5	5,151,189	4,848	10,041,216	292	80	205.0	SRR37195366	JBUZTC000000000
B534	*Pseudomonas lundensis*	58.7	5,085,529	4,803	10,127,430	299	36	391.7	SRR37195359	JBUZSV000000000
B622	*Pseudomonas lundensis*	58.6	5,233,968	4,890	10,826,814	310	50	241.9	SRR37195351	JBUZSO000000000
C124	*Pseudomonas lundensis*	58.7	4,944,793	4,618	9,974,690	303	68	206.1	SRR37195415	JBUZRZ000000000
D121	*Pseudomonas lundensis*	58.7	5,007,427	4,671	13,288,246	398	68	180.3	SRR37195458	JBUZQQ000000000
D123	*Pseudomonas lundensis*	58.5	5,314,332	5,003	11,063,356	312	71	254.8	SRR37195457	JBUZQP000000000
D125	*Pseudomonas lundensis*	58.4	5,260,895	4,950	11,813,052	337	76	205.0	SRR37195456	JBUZQO000000000
D131	*Pseudomonas lundensis*	58.5	5,037,565	4,682	11,229,298	334	51	255.3	SRR37195455	JBUZQN000000000
E135	*Pseudomonas lundensis*	58.5	5,196,234	4,903	8,501,090	245	39	295.4	SRR37195434	JBUZPU000000000
E233	*Pseudomonas lundensis*	58.4	5,055,800	4,698	11,388,014	338	69	174.5	SRR37195412	JBUZPP000000000
E322	*Pseudomonas lundensis*	58.7	4,834,098	4,492	7,782,358	241	27	488.2	SRR37195404	JBUZPH000000000
E324	*Pseudomonas lundensis*	58.5	5,096,759	4,751	10,308,254	303	65	279.7	SRR37195401	JBUZPF000000000
C314	*Pseudomonas mandelii*	58.4	7,789,315	7,307	12,951,578	249	79	300.0	SRR37195503	JBUZRC000000000
B333	*Pseudomonas paracarnis*	59.9	6,328,131	5,984	10,340,178	245	91	203.5	SRR37195466	JBUZTU000000000
B532	*Pseudomonas paracarnis*	59.7	6,487,213	6,008	9,206,120	213	64	256.9	SRR37195360	JBUZSW000000000
E311	*Pseudomonas paraveronii*	60.0	6,539,489	6,225	12,567,626	288	78	242.0	SRR37195410	JBUZPN000000000
E312	*Pseudomonas paraveronii*	59.8	7,202,758	6,999	10,925,008	228	151	147.3	SRR37195409	JBUZPM000000000
E314	*Pseudomonas paraveronii*	60.2	6,776,036	6,346	13,056,764	289	96	160.5	SRR37195407	JBUZPK000000000
E323	*Pseudomonas paraveronii*	60.3	6,460,188	6,138	11,814,890	274	87	171.3	SRR37195402	JBUZPG000000000
E332	*Pseudomonas paraveronii*	60.1	6,560,501	6,225	10,520,402	241	87	185.6	SRR37195399	JBUZPD000000000
A213	*Pseudomonas paraversuta*	58.2	5,388,624	4,948	10,170,614	283	71	239.6	SRR37195506	JBUZVI000000000
C112	*Pseudomonas paraversuta*	58.3	5,210,476	4,763	10,611,496	305	33	462.3	SRR37195422	JBUZSG000000000
C114	*Pseudomonas paraversuta*	58.1	5,199,540	4,728	9,878,708	285	46	414.6	SRR37195420	JBUZSE000000000
C125	*Pseudomonas paraversuta*	58.5	5,144,167	4,679	12,997,954	379	32	308.7	SRR37195527	JBUZRY000000000
C211	*Pseudomonas paraversuta*	58.4	5,099,603	4,624	13,283,338	391	31	349.8	SRR37195521	JBUZRS000000000
C213	*Pseudomonas paraversuta*	58.3	5,210,156	4,762	8,302,544	239	37	450.8	SRR37195519	JBUZRQ000000000
F121	*Pseudomonas paraversuta*	58.4	5,137,675	4,746	13,109,754	383	73	160.9	SRR37195396	JBUZPA000000000
B421	*Pseudomonas psychrophila*	57.0	5,643,929	5,243	10,569,892	281	79	220.4	SRR37195380	JBUZTO000000000
E1214	*Pseudomonas saxonica*	56.2	5,315,514	4,879	10,306,258	291	54	296.3	SRR37195439	JBUZPZ000000000
B631	*Pseudomonas shahriarae*	60.5	6,261,988	5,848	10,086,400	242	103	171.6	SRR37195428	JBUZSL000000000
C131	*Pseudomonas shahriarae*	60.4	6,339,454	5,918	12,346,054	292	72	234.3	SRR37195526	JBUZRX000000000
C225	*Pseudomonas shahriarae*	60.4	6,410,201	6,005	12,987,026	304	99	192.3	SRR37195512	JBUZRK000000000
C312	*Pseudomonas shahriarae*	60.1	6,647,336	6,204	10,169,478	229	93	209.2	SRR37195505	JBUZRE000000000
C324	*Pseudomonas shahriarae*	60.3	6,543,221	6,168	8,920,924	205	60	248.9	SRR37195500	JBUZQZ000000000
D221	*Pseudomonas shahriarae*	60.2	6,448,420	5,908	12,392,250	288	46	674.2	SRR37195447	JBUZQG000000000
A125	*Pseudomonas* sp.	59.9	7,717,410	7,317	8,618,960	168	101	212.3	SRR37195469	JBUZVP000000000
B233	*Pseudomonas* sp.	60.7	6,433,768	5,996	9,620,552	224	90	165.3	SRR37195478	JBUZUF000000000
B414	*Pseudomonas* sp.	57.8	5,518,585	5,164	10,643,256	289	92	129.1	SRR37195382	JBUZTQ000000000
B424	*Pseudomonas* sp.	58.1	5,614,558	5,269	11,257,884	301	84	193.5	SRR37195376	JBUZTL000000000
B611	*Pseudomonas* sp.	60.7	6,569,049	6,053	9,845,040	225	56	408.5	SRR37195357	JBUZST000000000
C311	*Pseudomonas* sp.	58.9	5,548,567	5,264	8,170,798	221	37	635.2	SRR37195507	JBUZRF000000000
C313	*Pseudomonas* sp.	59.0	5,899,062	5,557	12,977,620	330	52	576.7	SRR37195504	JBUZRD000000000
C321	*Pseudomonas* sp.	59.0	5,545,893	5,244	8,386,548	227	34	635.5	SRR37195502	JBUZRB000000000
E315	*Pseudomonas* sp.	55.5	4,843,332	4,407	12,144,062	376	40	267.9	SRR37195406	JBUZPJ000000000
B435	*Pseudomonas synxantha*	59.9	6,627,270	6,128	10,071,882	228	43	446.7	SRR37195372	JBUZTH000000000
C135	*Pseudomonas taetrolens*	58.5	4,704,621	4,348	10,341,688	330	19	633.5	SRR37195522	JBUZRT000000000
E313	*Pseudomonas veronii*	60.7	6,878,214	6,455	11,425,016	249	183	110.3	SRR37195408	JBUZPL000000000
A212	*Pseudomonas versuta*	58.0	5,268,647	4,882	9,252,450	263	21	517.5	SRR37195517	JBUZVJ000000000
A214	*Pseudomonas versuta*	58.0	5,382,781	4,987	8,632,204	241	43	321.9	SRR37195495	JBUZVH000000000
A224	*Pseudomonas versuta*	58.1	5,513,776	5,090	8,811,996	240	30	722.4	SRR37195403	JBUZVD000000000
B132	*Pseudomonas versuta*	57.7	5,529,173	5,232	9,202,504	250	38	447.8	SRR37195487	JBUZUN000000000
B621	*Pseudomonas versuta*	58.2	5,300,011	4,853	11,163,052	316	34	331.9	SRR37195352	JBUZSP000000000
B624	*Pseudomonas versuta*	57.9	5,508,123	5,133	9,616,928	262	39	377.5	SRR37195430	JBUZSN000000000
C331	*Pseudomonas versuta*	58.1	5,237,221	4,788	9,386,618	269	53	297.5	SRR37195498	JBUZQX000000000
C332	*Pseudomonas versuta*	58.3	5,299,312	4,800	10,928,690	309	36	286.7	SRR37195497	JBUZQW000000000
B111	*Pseudomonas weihenstephanensis*	57.4	4,963,563	4,686	9,617,926	291	34	439.0	SRR37195383	JBUZUU000000000
B112	*Pseudomonas weihenstephanensis*	57.4	4,961,992	4,689	7,348,146	222	36	327.4	SRR37195494	JBUZUT000000000
B131	*Pseudomonas weihenstephanensis*	57.3	5,284,157	4,835	9,529,604	271	46	246.3	SRR37195488	JBUZUO000000000
B212	*Pseudomonas weihenstephanensis*	57.4	4,958,145	4,686	8,775,102	265	40	327.4	SRR37195485	JBUZUL000000000
B332	*Pseudomonas weihenstephanensis*	57.2	5,243,786	4,816	12,769,494	365	44	646.2	SRR37195467	JBUZTV000000000
B334	*Pseudomonas weihenstephanensis*	57.4	5,202,255	4,810	7,473,154	215	51	313.8	SRR37195465	JBUZTT000000000
B431	*Pseudomonas weihenstephanensis*	57.4	5,213,654	4,819	9,299,938	268	46	429.3	SRR37195375	JBUZTK000000000
B525	*Pseudomonas weihenstephanensis*	57.4	5,217,434	4,835	12,374,304	356	50	313.8	SRR37195362	JBUZSY000000000
B531	*Pseudomonas weihenstephanensis*	57.3	5,147,500	4,746	9,650,212	281	55	455.6	SRR37195361	JBUZSX000000000
C134	*Pseudomonas weihenstephanensis*	57.2	4,827,227	4,501	11,129,856	346	25	564.5	SRR37195523	JBUZRU000000000
E321	*Pseudomonas weihenstephanensis*	57.3	4,756,051	4,435	12,694,504	400	24	641.5	SRR37195405	JBUZPI000000000
B422	*Rothia endophytica*	56.0	2,529,692	2,311	8,382,804	497	17	646.4	SRR37195379	JBUZTN000000000
C222	*Yersinia aleksiciae*	48.8	4,582,874	4,252	12,910,160	423	67	171.5	SRR37195515	JBUZRN000000000

## Data Availability

Both raw sequencing reads and genome sequences were deposited to GenBank under BioProject number PRJNA1420917 and their accession numbers are listed in Table 1.
